# Hypoxia inhibits TNF-α-induced TSLP expression in keratinocytes

**DOI:** 10.1371/journal.pone.0224705

**Published:** 2019-11-04

**Authors:** Naoyuki Tashiro, Ryosuke Segawa, Ryozo Tobita, Sanki Asakawa, Natsumi Mizuno, Masahiro Hiratsuka, Noriyasu Hirasawa

**Affiliations:** Laboratory of Pharmacotherapy of Life-Style Related Diseases, Graduate School of Pharmaceutical Sciences, Tohoku University, Sendai, Miyagi, Japan; University of Thessaly Faculty of Medicine, GREECE

## Abstract

The expression of thymic stromal lymphopoietin (TSLP), a cytokine which greatly contributes to the induction of type I allergy, is upregulated in chronic inflammation such as atopic dermatitis and psoriasis. As hypoxia in the epidermis is important for maintaining skin homeostasis, we examined the regulation of TSLP expression by hypoxic conditions in normal skin epithelial tissues. TNF-α-induced expression of TSLP in human keratinocyte HaCaT and in mouse keratinocyte PAM212 cell lines were inhibited under hypoxic condition (1% O_2_), although the mRNA expressions of TNF-α, IL-6, IL-8, MCP-1, and VEGF-A were not inhibited. Hypoxia-mimicking conditions, which include NiCl_2_, CoCl_2_, and DMOG, an inhibitor of 2-oxoglutarate-dependent enzymes, also selectively inhibited TNF-α-induced TSLP expression. These results suggested that inactivation of prolyl hydroxylase by hypoxia and hypoxia-mimicking conditions is involved in the repression of TNF-α-induced TSLP expression. Interestingly, the inhibition of TSLP production by hypoxic treatment was significantly reversed by treatment with the HIF-2α antagonist but not with the HIF-1α inhibitor. DMOG-induced inhibition of TSLP promoter activity was dependent on the -71 to +185 bp promoter region, suggesting that the binding of HIF-2 to hypoxia response element (HRE) in this region repressed the TSLP expression. These results indicated that hypoxia and hypoxia-mimicking conditions inhibited TSLP expression via HIF-2 and HRE-dependent mechanisms. Therefore, PHD and HIF-2α could be a new strategy for treatment of atopic dermatitis and psoriasis.

## Introduction

Thymic stromal lymphopoietin (TSLP) is considered a master switch for allergic inflammation [1]. TSLP is mainly produced by epithelial cells, and promotes Th2-type immune responses by activating on dendritic cells [2]. Activated dendritic cells express OX40L and promote differentiation of naive CD4 positive T cells into inflammatory Th2 cells, which aggravates allergies by releasing cytokines, such as IL-4, IL-5 and IL-13 [3]. TSLP production is induced by inflammatory cytokines, such as TNF-α [4], the activation of protease-activating receptors, toll-like receptors, and even by chemical compounds [5]. Both transcription factors NF-κB and AP-1 regulate the expression of human TSLP protein [6]. In addition, nuclear receptors, which include vitamin D [7] and retinoic acid receptors [8] also increased TSLP expression in mice.

Skin is divided into epidermis, dermis, and subcutaneous tissues, in the described order from outer to inner layers. The layers are supplied with oxygen from blood vessels in subcutaneous tissues [9]. Therefore, the oxygen concentration decreases from the inner layer to the epidermis. The oxygen concentrations in adult human and rodent epithelia is between 0.5–5% [9]. Hypoxia in the epidermis is important for maintaining skin homeostasis via inactivation of proline hydroxylase (PHD), a sensor of oxygen concentration. PHD enhances HIF-α degradation via hydroxylation of HIFs in normoxia, but the hydroxylation is inhibited in a low oxygen environment. HIFs positively or negatively regulate the expression of various proteins via binding to hypoxia response element (HRE) [10, 11]. For instance, HIFs enhance the expression of a barrier protein filaggrin in epithelial cells [10, 12]. On the other hand, HIFs downregulate several proteins such as tissue factor pathway inhibitor (TFPI) through binding to negative HREs sequences, which were identified as 5′-GACATG-3′ [13] and 5′-AAACAGGA-3′ in the human breast cancer MCF-7 cell line [14].

Recently, it is reported that PHD inhibition reduced TNF-α-induced production of inflammatory chemokines in keratinocytes and ameliorated allergic contact dermatitis [15]. Therefore, we hypothesized that hypoxia of epidermal tissue control TSLP expression, too.

In this research, using human keratinocyte HaCaT and mouse keratinocyte PAM212 cell lines, we examined whether TSLP expression was regulated by hypoxia and identified the molecular mechanisms involved in the process by using PHD inhibitors [16].

## Materials and methods

### Materials

Human and mouse recombinant TNF-α proteins were purchased from Peprotech (Rocky Hill, NJ). Nickel chloride (NiCl_2_), cobalt chloride (CoCl_2_), and magnesium chloride (MgCl_2_) were purchased from Wako Pure Chemical Ind (Osaka, Japan). Dimethyloxalylglycine (DMOG) was purchased from Sigma Aldrich (St. Louis, MO). PX-478, an HIF-1α inhibitor, and N-(3-Chloro-5-fluorophenyl)-4-nitrobenzo[c][1,2,5]oxadiazol-5-amine, an HIF-2α antagonist, were purchased from Calbiochem (San Diego, CA) and Sigma Aldrich, respectively. Dulbecco´s modified eagle medium, (DMEM), minimum essential medium α, (MEM-α) and keratinocyte growth medium, (KGM; KGM-Gold^TM^ Bullet kit) were purchased from Nissui Pharmaceutical Co (Tokyo, Japan), Life Technologies (Grand Island, NY) and Lonza (Walkersville, MD), respectively. FBS was purchased from Biowest (Miami, FL). Antibodies used were anti-HIF-1α (Santa Cruz Biotechnology, # sc-10790), anti-HIF-2α (Novus Biologicals, # NB100-122SS), and anti-actin antibodies (Santa Cruz Biotechnology, # sc-1616). TSLP promoter reporter plasmid [17] was a gift from Dr. Tamari, Institute of Physical and Chemical Research, Japan. Cytomegalovirus (CMV) and thymidine kinase (TK) reporter plasmids were purchased from Promega (Madison, WI).

### Cell culture and stimulation

The human keratinocyte HaCaT (Dr. N.E. Fusenig, German Cancer Research Center) and the mouse keratinocyte PAM212 cell lines (Dr. Yuspa, National Institutes of Health) were used in this study. HaCaT cells and PAM212 cells were cultured at 37°C under a humidified atmosphere of 5% CO_2_−95% air in 10% FBS-DMEM and 10% FBS-MEM-α, respectively. A total of 0.7 × 10^5^ HaCaT cells/ml in KGM were seeded into 24 well plates. Two days later, fresh medium was added. In the next day, the cells were incubated for 8 h or indicated time under hypoxia (1% O_2_-5% CO_2_-94% N_2_) or normoxia (5% CO_2_-95% Air), or pretreated with NiCl_2,_ CoCl_2_ or DMOG at the indicated concentrations for 2 to 24 h, and then stimulated with TNF-α (100 ng/ml). After HaCaT cells were pretreated under hypoxia, the cells were stimulated by TNF-α under hypoxia. PAM212 cells were seeded in 10% FBS-MEM-α in 24 well plates. Under 80% confluent conditions, PAM212 cells were treated the same way as with HaCaT cells.

### Quantitative real-time PCR

Total RNA of the stimulated cells was extracted with RNAiso Plus (Takara, Shiga, Japan) according to the manufacturer's instructions. Total RNA was reverse-transcribed using the PrimeScript RT reagent kit (Takara) and then PCR-amplified with Takara PCR Thermal Cycler Dice (Takara) using SYBR Premix Ex Taq II (Takara). The following oligonucleotides, F (forward) and R (reverse), were used for PCR: human β2-microglobulin F: 5′-CTATCCAGCGTACTCCAAAG-3′ and R: 5′-GAAAGACCAGTCCTTCCTGA-3′, mouse β2-microglobulin F: 5′-TTCTGGTGCTTGTCTCACTGA-3′ and R: 5′-CAGTATGTTCGGCTTCCCATTC-3′, human TSLP F: 5′-GATTACATATATGAGTGGGAC-3′ and R: 5′-TTCATTGCCTGAGTAGCAT-3′, human Vascular Endothelial Growth Factor A (human VEGF-A) F: 5´-AGGCCAGCACATAGGAGAGA-3´ and R: 5´-TTTCTTGCGCTTTCGTTTTT-3´, IL-8 F: 5´-CAATCCTAGTTTGATACTCCC-3´ and R: 5´-AATTACTAATATTGACTGTGGAG-3´, Filaggrin F: 5´-CAATCAGGCACTCATCACAC-3´ and R: 5´- ACTGTTAGTGACCTGACTACC-3´, IL-6 F: 5´-AACAACCTGAACCTTCCAAAGA-3´ and R: 5´-TCAAACTCCAAAAGACCAGTGA-3´, Monocyte Chemotactic Protein-1 (MCP-1) F: 5′-GATCTCAGTGCAGAGGCTCG-3′ and R: 5′-TGCTTGTCCAGGTGGTCCAT-3´, TNF-α F: 5′-TGTAGCCCATGTTGTAGCAAC-3′ and R: 5´-TTGAAGAGGACCTGGGAGTAGA-3´, mouse TSLP F: 5´-CGAGCAAATCGAGGACTGTGAG-3´ and R: 5´-GCAGTGGTCATTGAGGGCTTC-3´, mouse Vascular Endothelial Growth Factor (mouse VEGF) F: 5´-CTGGCTTTACTGCTGTACCTC-3´ and R: 5´-CATGGTGATGTTGCTCTCTGAC-3´.

### MTT assay

After the stimulation with TNF-α, the medium was changed to KGM (250 μl/well), and MTT was added (25 μl/well). Four hours later, the medium was removed and DMSO was added into each well (250 μl/well) to dissolve the insoluble formazan product. Absorbance at 595 nm was determined.

### Western blot

After the treatment with Hypoxia, NiCl_2_ and CoCl_2_ for 8 h, the cells were lysed in the lysis buffer that contained protease inhibitor cocktail (20 mM HEPES buffer including 1% (v/v) Triton-X 100, 10% (v/v) glycerol, 1 mM EDTA, 10 μg/ml leupeptin, 10 μg/ml phenylmethylsulfonyl fluoride, 50 mM NaF, 1 mM Na_3_VO_4_ and 2.5 mM p-nitrophenyl phosphate). HIF-1α, HIF-2α and actin were detected by Western blot.

### Construction of reporter plasmids for luciferase assay

We used the reporter plasmid which includes -4102 to +185 bp TSLP promoter region. The deletion of TSLP promoter region was performed by using KOD -Plus- Mutagenesis Kit (TOYOBO, Tokyo, Japan). The used primers were as follows; (-4102 bp forward) 5′-GCCCTGTAGGAGAAAGACACTGGTATC-3′, (-3732 bp forward) 5′-TTTTCTTAATCCAAAGAGACAGATCTCCC-3′, (-3214 bp forward) 5′-CAAACTACGTATGCAGATACTGTTCAC-3′, (-1330 bp forward) 5′-AGGAACTTCCCAAGGACCAG-3′ and (reverse) 5′-TATCGATAGAGAAATGTTCTGGCACCTGC-3′, (Deletion -71 to +185 bp forward) 5′-CTCGAGATCTGCGATCTAAGTAA-3′ and (Deletion -71 to +185 bp reverse) 5′-CCTTTATAGAATTCTGAATTGATGATGTG-3′.

### Luciferase assay

The Dual-luciferase reporter assay system (Promega Corporation, Madison, WI) was used for the measurement of Firefly and Renilla luciferase activities. The measurements were conducted according to the manufacturer’s protocols. HaCaT cells (0.7 × 10^5^ cells/ml) were seeded onto 24-well plates. Two days later, the culture medium was removed and the cells were transfected with TSLP promoter reporter plasmid (250 ng/well) or HRE reporter plasmids along with CMV (10 ng/well) or TK (50 ng/well) reporter plasmids for 24 h by X-tremeGENE HP DNA Transfection Reagent (Roche). HaCaT cells were then lysed with 100 μl of lysis buffer (Promega Corporation, Madison, WI, USA), and 10 μl of the lysate was used for measurement of Firefly and Renilla luciferase activities.

### Statistical analysis

All data were indicated as the mean of values ± standard error of the mean (SEM). Comparisons of groups were evaluated by Student’s t-test. Multiple comparisons were performed with Bonferroni, Dunnett’s and Student-Newman-Keuls tests.

## Results

### Hypoxia selectively inhibits TNF-α-induced TSLP expression

**Fig 1:**
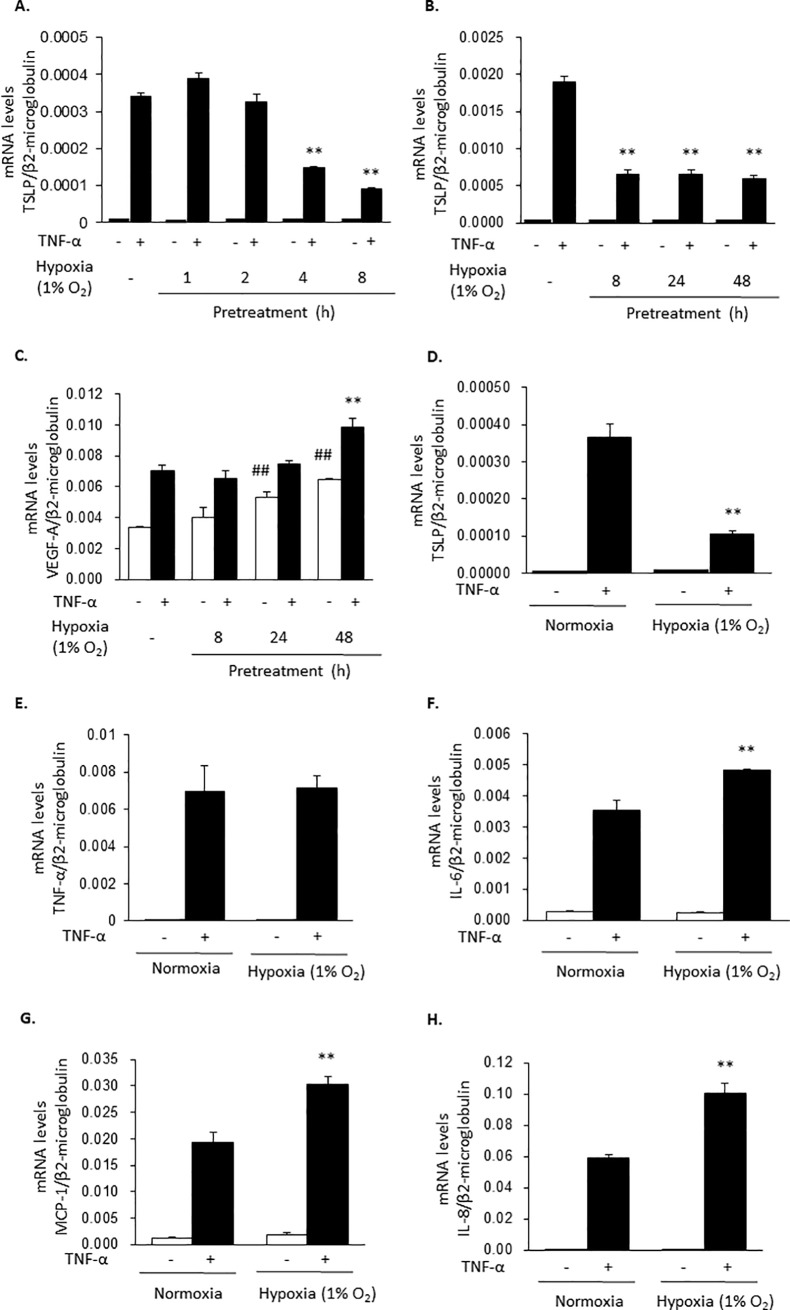
Hypoxia selectively inhibits TNF-α-induced TSLP expression. HaCaT cells were pretreated under a hypoxic condition (1% O_2_) for 8 h or indicated time and then stimulated with TNF-α (100 ng/ml) for 2 h. The mRNA levels for TSLP (A, B and D), VEGF-A (C), TNF-α (E), IL-6 (F), MCP-1 (G) and IL-8 (H) were determined by real time PCR. Data are indicated as means ± SEM from 3 samples. Statistical significance: * p < 0.05, ** p < 0.01 vs. corresponding TNF-α group in normoxia.

The stimulation of HaCaT cells with TNF-α (100 ng/ml) induced the expression of TSLP mRNA at 2 h. To analyze the effects of hypoxia on TSLP mRNA levels, HaCaT cells were incubated for 8 h or indicated time under hypoxia (1% O_2_) or normoxia and then stimulated with TNF-α for 2 h. As shown in Fig 1A, 1B and 1D, hypoxia treatment significantly reduced TSLP mRNA expression. In contrast, VEGF-A, TNF-α, IL-6, MCP-1, and IL-8 mRNA levels were maintained, or rather increased, by the hypoxic treatment (Fig 1C and 1E–1H), suggesting that TSLP expression was selectively inhibited by the hypoxia condition.

### NiCl_2_ and CoCl_2_ inhibit TNF-α-induced TSLP expression

**Fig 2:**
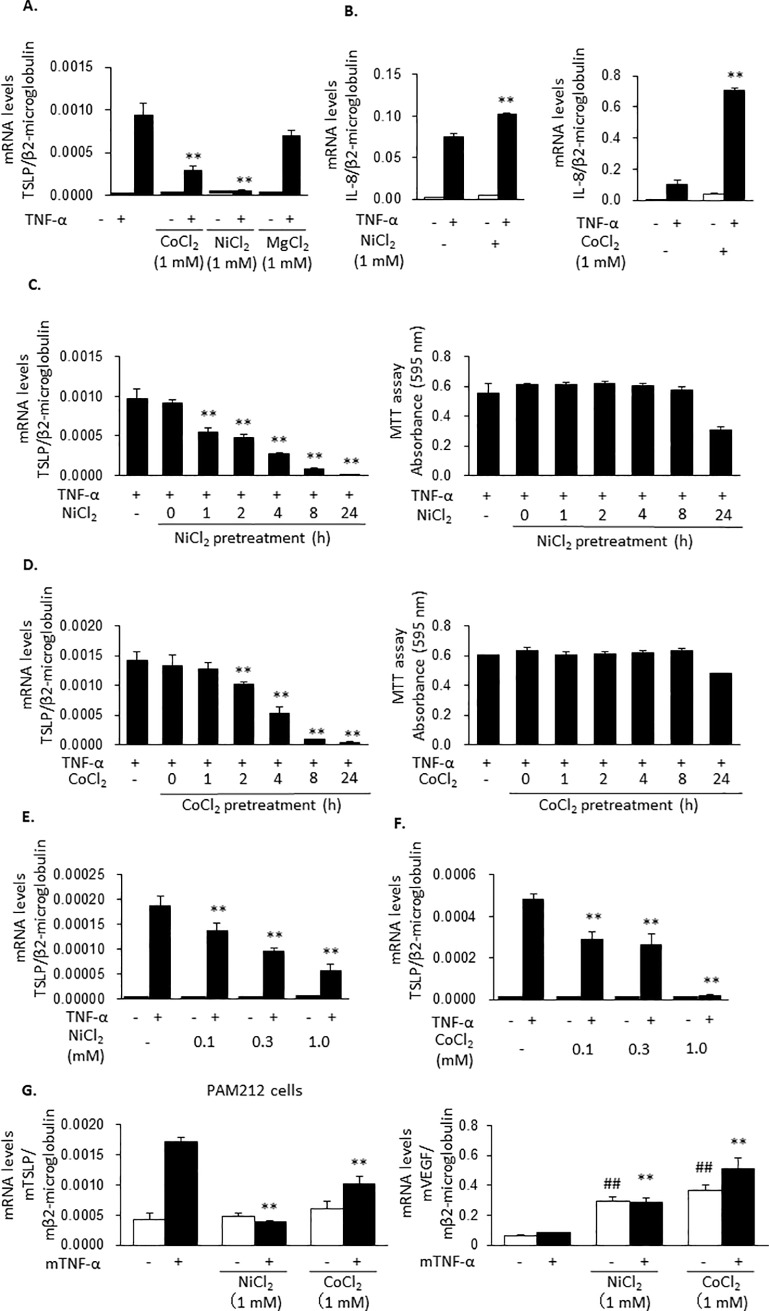
NiCl_2_ and CoCl_2_ inhibit TNF-α-induced TSLP expression. (A and B) HaCaT cells were pretreated with NiCl_2_, CoCl_2_ and MgCl_2_ (1 mM) for 24 h and stimulated with TNF-α (100 ng/ml) for 2 h. The expression of mRNAs for TSLP (A) and IL-8 (B) were determined. (C, D) HaCaT cells were pretreated for 8 h with NiCl_2_ and CoCl_2_ (1 mM) for 0, 1, 2, 4, 8, 24 h and then stimulated with TNF-α (100 ng/ml) for 2 h. The expression of TSLP mRNA was determined (left panels). Cytotoxic effects by NiCl_2_ and CoCl_2_ were evaluated by MTT assay (right panels). (E and F) HaCaT cells were pretreated by NiCl_2_ (E) or CoCl_2_ (F) at the concentrations of 0.1, 0.3 and 1 mM for 8 h and stimulated by TNF-α (100 ng/ml) for 2 h. (G) PAM212 cells were pretreated by NiCl_2_ and CoCl_2_ (1 mM) for 8 h and stimulated with mouse TNF-α (100 ng/ml) for 2 h. The levels of mRNA for TSLP (left panel) and VEGF (right panel) were determined. Data are indicated as means ± SEM from 3 samples. Statistical significance: * p < 0.05, ** p < 0.01, *** p < 0.001 vs. TNF-α only. ## p < 0.01, ### p < 0.001 vs. control.

To identify the molecular mechanisms by which hypoxia treatment inhibited TNF-α-induced TSLP expression, hypoxia conditions were mimicked with NiCl_2_ and CoCl_2_. Meanwhile, MgCl_2_ was used as a divalent cation control. TNF-α-induced TSLP mRNA expression in HaCaT cells was inhibited by 1 mM NiCl_2_ and 1 mM CoCl_2_, but not by 1 mM MgCl_2_ (Fig 2A). Similarly to the results obtained under the effects of hypoxia, NiCl_2_ and CoCl_2_ upregulated TNF-α-induced IL-8 expression (Fig 2B). Additionally, the inhibitory effects of NiCl_2_ and CoCl_2_ increased with the pre-incubation time until 8 h, without affecting cell viability, (Fig 2C and 2D) and with higher concentrations (Fig 2E and 2F). Similar results were obtained in the mouse keratinocyte PAM212 cell line. Namely, NiCl_2_ and CoCl_2_ significantly inhibited TNF-α-induced TSLP expression and TSLP protein (Fig 2G left panel, S1 Fig). Also, both NiCl_2_ and CoCl_2_ induced the expression of VEGF, indicating that these chemicals induced hypoxia mimicking conditions at the used concentrations (Fig 2G right panel).

### DMOG, an inhibitor of 2-oxoglutarate-dependent enzymes, inhibits TNF-α-induced TSLP expression

**Fig 3:**
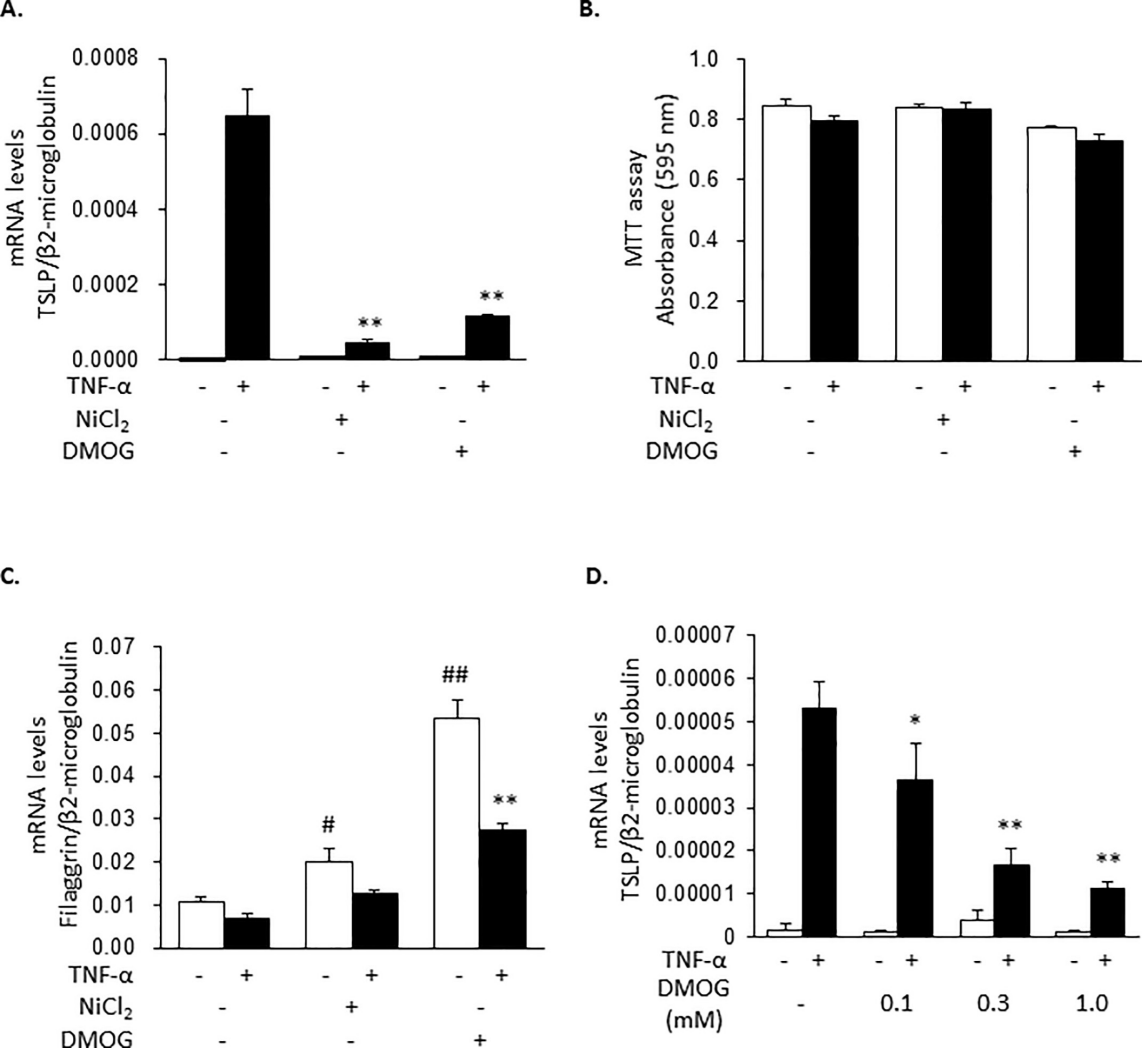
DMOG inhibits TNF-α-induced TSLP expression. (A to C) HaCaT cells were pretreated with NiCl_2_ (1 mM) and DMOG (1 mM) for 8 h and then stimulated with TNF-α (100 ng/ml) for 2 h. The expressions of mRNA for TSLP (A) and filaggrin (C) were determined. Cytotoxic effects of NiCl_2_ and DMOG were evaluated by MTT assay (B). (D) HaCaT cells were pretreated with DMOG at the indicated concentrations for 8 h and then stimulated by TNF-α (100 ng/ml) for 2 h. Data are indicated as means ± SEM from 3 samples. Statistical significance: * p < 0.05, ** p < 0.01, *** p < 0.001 vs. corresponding TNF-α only. # p < 0.05, ### p < 0.001 vs. control.

To confirm whether the inhibitory actions of hypoxia on TSLP expression were mediated by inhibition of PHD, the effects of DMOG, an inhibitor of 2-oxoglutarate-dependent enzymes which include PHD, were examined. As shown in Fig 3A, DMOG inhibited TNF-α-induced TSLP expression to the same extent as NiCl_2_ without affecting viability (Fig 3B). Additionally, both DMOG and NiCl_2_ increased filaggrin expression (Fig 3C), suggesting that these treatments resulted in the inhibition of PHD and the activation of HIFs-dependent pathway. The effects of DMOG were also dependent on its concentration (Fig 3D).

### Hypoxia and hypoxia mimicking conditions inhibit TNF-α-induced TSLP expression via HIF-2α but not HIF-1α

**Fig 4:**
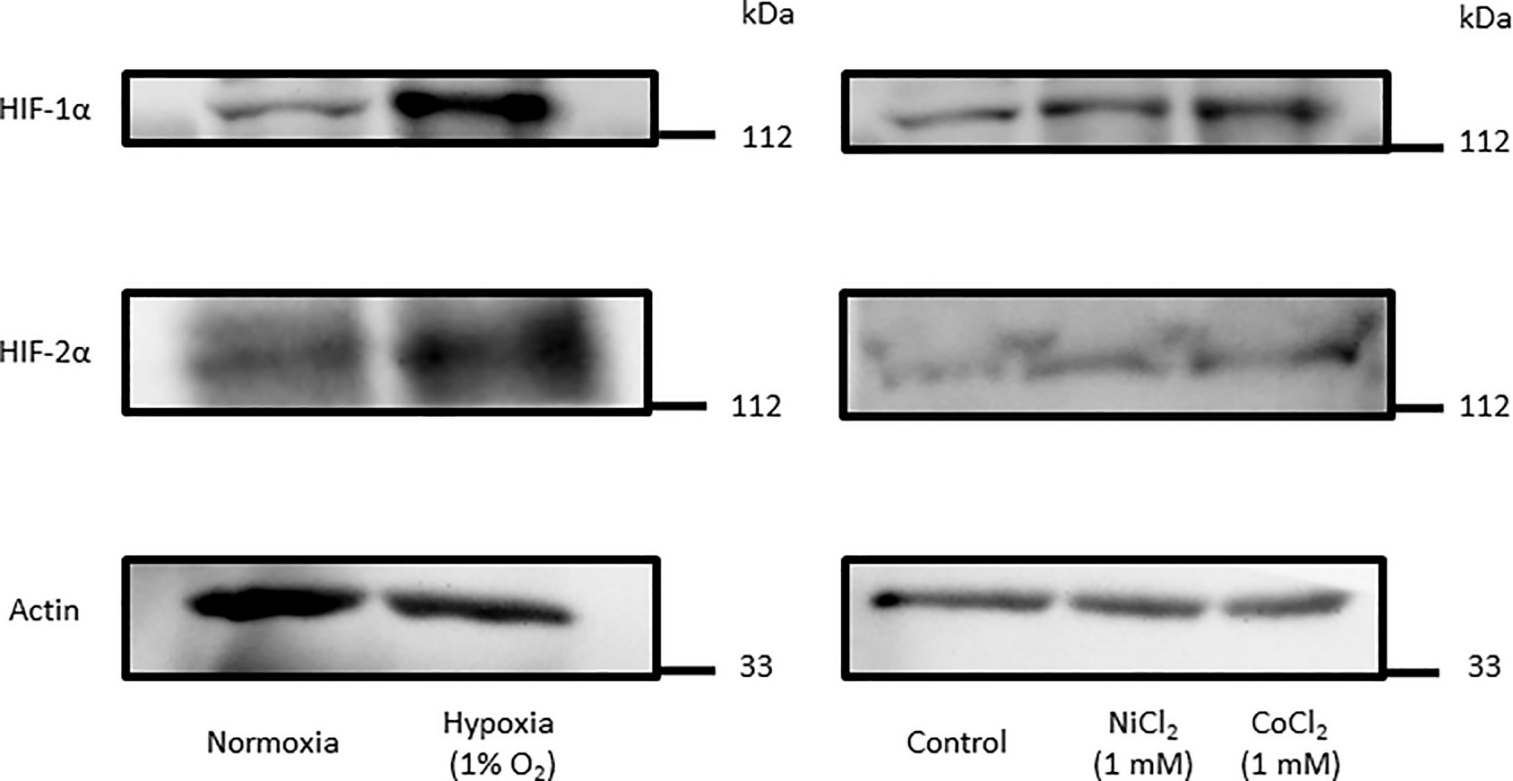
Hypoxia, NiCl_2_ and CoCl_2_ increased levels of HIF-1α and HIF-2α proteins. HaCaT cells were treated with hypoxia (1% O_2_), NiCl_2_ and CoCl_2_ (1 mM) for 8 h. The expression of HIF-α and actin proteins were determined by western blot.

**Fig 5:**
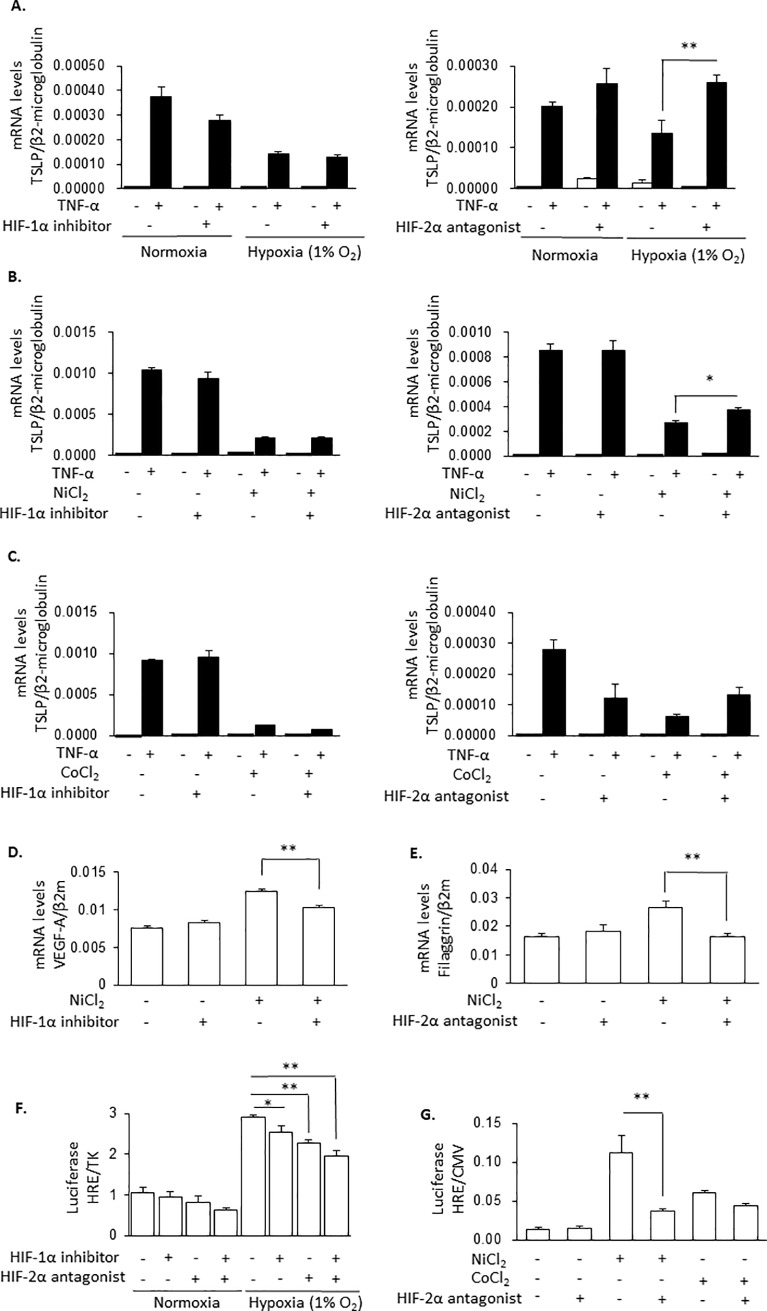
Hypoxia and hypoxia-mimicking conditions inhibit TNF-α-induced TSLP expression via HIF-2α but not HIF-1α expression. (A to E) HaCaT cells were pretreated by HIF-1α inhibitor (10 μM) and HIF-2α antagonist (100 μM) in normoxic and hypoxic condition (1% O_2_) (A) or with 1 mM NiCl_2_ (B) and 1 mM CoCl_2_ (C) for 8 h and stimulated by TNF-α (100 ng/ml) for 2 h. TSLP mRNA levels were determined. (D and E) HaCaT cells treated by NiCl_2_ (1 mM) and HIF-1α inhibitor (10 μM) (D) or HIF-2α antagonist (100 μM) (E) for 10 h. The levels of mRNA for TSLP (A to C), VEGF-A (D) and filaggrin (E) were determined. (F and G) HaCaT cells were transiently transfected with HRE luciferase reporter constructs, and 24 h later, incubated in normoxia and hypoxia (1% O_2_) for 8 h (F) or treated with 1 mM NiCl_2_ and 1 mM CoCl_2_ for 8 h (G) in the presence or absence of HIF-1α inhibitor (10 μM) and HIF-2α antagonist (100 μM). Firefly luciferase activity was normalized to Renilla luciferase activity. Data are indicated as means ± SEM from 3 or 4 samples. Statistical significance: * p < 0.05, ** p < 0.01 between the indicated groups.

Treatment of HaCaT cells under hypoxia condition or, in presence of NiCl_2_ and CoCl_2_ for 8 h increased HIF-1α and HIF-2α protein levels (Fig 4). Therefore, we next examined the roles of HIF-1 and HIF-2 proteins on inhibition of TSLP expression under hypoxia by using a HIF-1 inhibitor and a HIF-2 antagonist. The inhibition of TNF-α-induced TSLP expression by hypoxia (Fig 5A), by NiCl_2_ (Fig 5B) or CoCl_2_ (Fig 5C) was not revered with the HIF-1α inhibitor. On the other hand, their effects were all antagonized by HIF-2α antagonist (Fig 5A–5C, right panels). Higher concentrations of the HIF-1 inhibitor also did not recover the NiCl_2_-induced inhibition of TSLP expression (S2 Fig). In contrast, NiCl_2_-induced expressions of VEGF-A (Fig 5D) and filaggrin (Fig 5E) were significantly inhibited by the HIF-1 inhibitor and the HIF-2 antagonist, respectively. The HIF-1 inhibitor and the HIF-2 antagonist inhibited hypoxia-induced HRE activity and showed additive effects (Fig 5F). NiCl_2_- and CoCl_2_-induced HRE activities were also significantly inhibited by HIF-2 antagonist (Fig 5G). These results suggested that the inhibitory actions of hypoxia and hypoxia-mimicking conditions (NiCl_2_ and CoCl_2_) on TSLP expression were mediated mainly by HIF-2α.

### Negative HRE element is located between -71 and +185 bp of TSLP promoter

**Fig 6:**
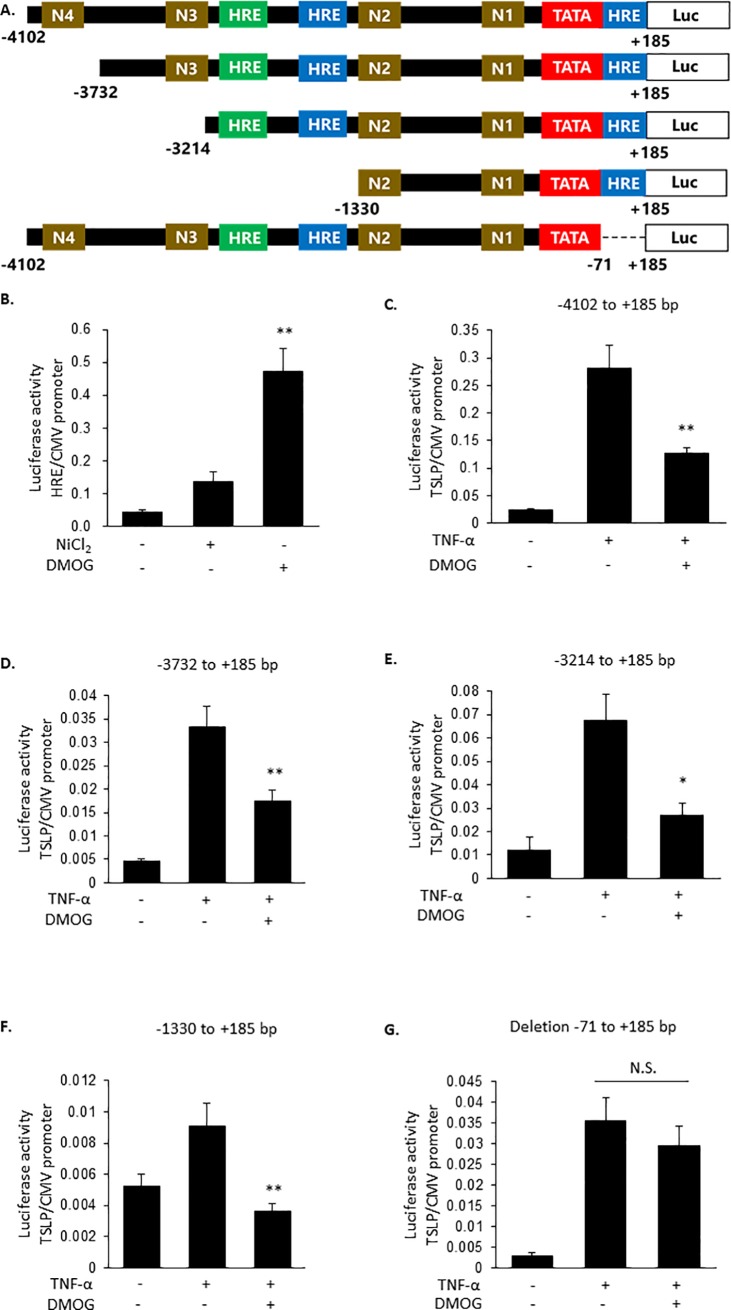
The region -71 and +185 bp of TSLP promoter is involved in DMOG-induced repression of TSLP expression. (A) Schematic diagram of TSLP reporter plasmid used in this study. HRE: putative hypoxia responsive element, N1 to N4: NF-κB binding sites, Luc: Luciferase. (B) HaCaT cells were transiently transfected with HRE luciferase reporter constructs, and 24 h later, stimulated with NiCl_2_ and DMOG (1 mM) for 8 h. Firefly luciferase activity was normalized to Renilla luciferase activity. (C-G) HaCaT cells were transiently transfected with TSLP promoter luciferase reporter constructs indicated in A. Twenty four hours later, the cells were treated for 8 h with DMOG (1 mM) and then stimulated with TNF-α (100 ng/ml) for 8 h. Firefly luciferase activity was normalized to Renilla luciferase activity. Data are indicated as means ± SEM from 3 or 4 samples. Statistical significance: * p < 0.05 vs. corresponding TNF-α control, N.S.: Not significant.

Finally, we examined whether HIF-2 exerted its inhibitory effect via binding to HRE. The reporter assay using HRE reporter plasmid showed that DMOG increased promoter activity more potently than NiCl_2_ (Fig 6B). Therefore, DMOG was used in the following experiments. In the promoter region (-4102 to +185 bp) of TSLP gene, 4 NF-κB sites (N1-N4), which played important roles in the expression of TSLP [18, 19], and 3 negative HRE consensus sites were identified. To determine whether HIF-2 inhibited TSLP expression via binding to HRE, we created four reporter plasmids in which NF-κB sites or putative HRE sites were deleted (Fig 6A). As consistent with TSLP expression (Fig 3), DMOG inhibited the promoter activity of -4102 to +185 bp region (Fig 6C). The deletion of N4 and N3 (Fig 6D and 6E), and the deletion of HRE between -3214 to -1330 bp (Fig 6F) did not alter the effect of DMOG. However, DMOG failed to inhibit the reporter activity of when the -71 to +185 bp (-4102 to -72 bp) region was deleted (Fig 6G). These results indicated that HIF-2 induced inhibition of TSLP promoter activity was dependent on the region between -71 and +185 bp, which could probably contain an HRE (S3 Fig).

## Discussion

In this study, we determined that hypoxia and hypoxia-mimicking conditions selectively attenuated TNF-α-induced TSLP expression. Our results also suggested that HIF-2α, but not HIF-1α, inhibited the expression of TSLP through binding to an HRE site.

First, we evidenced that the treatment of the cells under hypoxia for 8 h suppressed TNF-α-induced TSLP mRNA expression but not the expressions of TNF-α, IL-6, MCP-1, VEGF-A and IL-8 mRNA in the human epidermal keratinocyte HaCaT cell line (Fig 1). Similar results were obtained with PAM212 cells. NiCl_2_, CoCl_2_ and DMOG also selectively inhibited TNF-α-induced TSLP expression (Figs 2 and 3). In addition, in these conditions, HIF-1α and HIF-2α protein levels were increased (Fig 4). These findings indicates that the inhibition of TSLP expression in hypoxia was due to the inhibition of PHD.

A previous investigation noted that inhibition of PHD stabilized HIF-1α and HIF-2α proteins and induced HIF-dependent regulation of transcription. In this study, HIF-α proteins were detected in spite of under normoxia (Fig 4). It is reported that, in HaCaT cells, the expressions of HIF-α proteins were detected by increasing cell density in normoxia [20]. Even so, the levels of both HIF-1α and HIF-2α proteins were further increased by the treatment with hypoxia and hypoxia mimics (Fig 4). It was also suggested that HIF-1α and HIF-2α have different physiological functions [21]. The different involvements of HIF-1α and HIF-2α might depend on the target gene and the tissue-specific expression of the isoforms [21]. In this study, by using HIF inhibitors, it was proposed that HIF-2α mainly played a role in the down-regulation of TSLP expression.

Other studies reported that HIF-1α and HIF-2α negatively regulated the transcription of Fas-associated death domain protein (FADD) [22], cystic fibrosis transmembrane conductance regulator [23] and nucleoside transporter 2 [24] in epithelial cells, and of tissue factor pathway inhibitor (TFPI) in endothelial cells [25] and breast cancer MCF-7 cell line [14]. The negative regulation was mediated by binding to a specific HRE, for instance, to the region containing 5′-GACATG-3′ [13] and 5′-AAACAGGA-3′ [14] sequences. In the promoter region of human TSLP, the 5′-GACATG-3′ sequence was located at -2389 to -2384 bp and -62 to -57 bp regions; meanwhile, the 5′-AAACAGGA-3′ sequence was detected in the -3144 to -3137 bp region. The inhibitory action of DMOG on TSLP promoter activity was observed in the reporter gene, between the -1330 to +185 bp region, but disappeared when deleting the -71 to +185 bp region (Fig 6). These findings suggested that the putative HRE site at -62 to -57 bp might be involved in the suppression.

The expression of a barrier protein, filaggrin, in keratinocytes was upregulated under a hypoxic condition [12] (Fig 3C) and inhibited by inflammatory stimuli, such as TNF-α [26] (Fig 3C). TSLP expression was induced by TNF-α [4] and inhibited under hypoxia (1% O_2_). Since the oxygen concentration in adult human and rodent epithelia is reported as 0.5–5% [9], it is likely that TSLP expression is maintained at a low level in normal skin epithelial tissues. Thus, it is important to keep hypoxia in epithelial tissues to avoid the onset and exacerbation of allergy via downregulating TSLP and enhancing filaggrin expressions [12]. Conversely, the disruption of hypoxia in the epidermis by acute and/or chronic inflammation would result in exacerbation of allergic inflammation. This might be one of the mechanisms by which chronic inflammation exacerbates atopic dermatitis.

In conclusion, this study revealed that TSLP expression was downregulated in hypoxia via HIF-2 and the -62 to -57 bp TSLP promoter region-dependent ways. Therefore, PHD and HIF-2 in the epidermis might be a novel therapeutic approach for treating atopic dermatitis.

## Supporting information

S1 Fig NiCl_2_ inhibits TNF-α-induced TSLP production. PAM212 cells were incubated for 24 h in medium containing TNF-α in the presence or absence of NiCl_2_ at the indicated concentrations. The concentrations of TSLP in the medium were determined by Enzyme-linked immunoassay (ELISA) and cell viability was determined by the MTT assay. Data are indicated as means ± SEM from 4 samples. Statistical significance: ** p < 0.01 vs. corresponding TNF-α control.
(PDF)

S2 Fig Effects of HIF-1α inhibitor at higher concentrations on TNF-α-induced TSLP expression. HaCaT cells were pretreated with HIF-1α inhibitor (25 μM, 50 μM) and NiCl_2_ (1 mM) for 8 h and stimulated with TNF-α (100 ng/ml) for 2 h. Data are indicated as means ± SEM from 3 samples.
(PDF)

S3 Fig The -71 to +185 bp region in the TSLP promoter sequence. Putative HRE site, 5′-GACATG-3′, is indicated. HRE was searched by the TRANSFAC program (Match -1.0 Public).
(PDF)

## References

[1] He R, Geha RS. Thymic stromal lymphopoietin. Ann N Y Acad Sci. 2010; 1183: 13–24. 10.1111/j.1749-6632.2009.05128.x 20146705 PMC2895428

[2] Omori-Miyake M, Ziegler SF. Mouse models of allergic diseases: TSLP and its functional roles. Allergol Int. 2012; 61: 27–34. 10.2332/allergolint.11-RAI-0374 22270069

[3] Zhou B, Comeau MR, De Smedt T, Liggitt HD, Dahl ME, Lewis DB, et al Thymic stromal lymphopoietin as a key initiator of allergic airway inflammation in mice. Nat Immunol. 2005; 6: 1047–53. 10.1038/ni1247 16142237

[4] Segawa R, Shigeeda K, Hatayama T, Dong J, Mizuno N, Moriya T, et al EGFR transactivation is involved in TNF-α-induced expression of thymic stromal lymphopoietin in human keratinocyte cell line. J Dermatol Sci. 2018; 89: 290–298. 10.1016/j.jdermsci.2017.12.008 29279286

[5] Segawa R, Hirasawa N. Exacerbation of allergic diseases by chemicals: role of TSLP. J Pharmacol Sci. 2014; 124: 301–6. 10.1254/jphs.13r16cp 24599138

[6] Tsilingiri K, Fornasa G, Rescigno M. Thymic Stromal Lymphopoietin: To Cut a Long Story Short. Cell Mol Gastroenterol Hepatol. 2017; 3: 174–182. 10.1016/j.jcmgh.2017.01.005 28275684 PMC5331833

[7] Li M, Hener P, Zhang Z, Kato S, Metzger D, Chambon P. Topical vitamin D3 and low-calcemic analogs induce thymic stromal lymphopoietin in mouse keratinocytes and trigger an atopic dermatitis. Proc Natl Acad Sci U S A. 2006; 103: 11736–41. 10.1073/pnas.0604575103 16880407 PMC1544239

[8] Hatayama T, Segawa R, Mizuno N, Eguchi S, Akamatsu H, Fukuda M, et al All-*Trans* Retinoic Acid Enhances Antibody Production by Inducing the Expression of Thymic Stromal Lymphopoietin Protein. J Immunol. 2018; 200: 2670–2676. 10.4049/jimmunol.1701276 29500243

[9] Evans SM, Schrlau AE, Chalian AA, Zhang P, Koch CJ. Oxygen levels in normal and previously irradiated human skin as assessed by EF5 binding. J Invest Dermatol. 2006; 126: 2596–606. 10.1038/sj.jid.5700451 16810299

[10] O'Shaughnessy RFL, Brown SJ. Insight from the air-skin interface. J Invest Dermatol. 2015; 135: 331–333. 10.1038/jid.2014.457 25573043

[11] Befani C, Mylonis I, Gkotinakou IM, Georgoulias P, Hu CJ, Simos G, et al Cobalt stimulates HIF-1-dependent but inhibits HIF-2-dependent gene expression in liver cancer cells. Int J Biochem Cell Biol. 2013; 45: 2359–68. 10.1016/j.biocel.2013.07.025 23958427 PMC3855297

[12] Wong WJ, Richardson T, Seykora JT, Cotsarelis G, Simon MC. Hypoxia-inducible factors regulate filaggrin expression and epidermal barrier function. J Invest Dermatol. 2015; 135: 454–461. 10.1038/jid.2014.283 24999590 PMC4286527

[13] Cui XY, Tinholt M, Stavik B, Dahm AE, Kanse S, Jin Y, et al Effect of hypoxia on tissue factor pathway inhibitor expression in breast cancer. J Thromb Haemost. 2016; 14: 387–96. 10.1111/jth.13206 26598923

[14] Cui XY, Skretting G, Tinholt M, Stavik B, Dahm AEA, Sahlberg KK, et al A novel hypoxia response element regulates oxygen-related repression of tissue factor pathway inhibitor in the breast cancer cell line MCF-7. Thromb Res. 2017; 157: 111–116. 10.1016/j.thromres.2017.07.013 28734156

[15] Manresa MC, Smith L, Casals-Diaz L, Fagundes RR, Brown E, Radhakrishnan P, et al Pharmacologic inhibition of hypoxia-inducible factor (HIF)-hydroxylases ameliorates allergic contact dermatitis. Allergy. 2019; 74: 753–766. 10.1111/all.13655 30394557

[16] Salnikow K, Donald SP, Bruick RK, Zhitkovich A, Phang JM, Kasprzak KS. Depletion of intracellular ascorbate by the carcinogenic metals nickel and cobalt results in the induction of hypoxic stress. J Biol Chem. 2004; 279: 40337–44. 10.1074/jbc.M403057200 15271983

[17] Harada M, Hirota T, Jodo AI, Hitomi Y, Sakashita M, Tsunoda T. Thymic stromal lymphopoietin gene promoter polymorphisms are associated with susceptibility to bronchial asthma. Am J Respir Cell Mol Biol. 2011; 44: 787–93. 10.1165/rcmb.2009-0418OC 20656951 PMC3159073

[18] Redhu NS, Saleh A, Halayko AJ, Ali AS, Gounni AS. Essential role of NF-κB and AP-1 transcription factors in TNF-α-induced TSLP expression in human airway smooth muscle cells. Am J Physiol Lung Cell Mol Physiol. 2011; 300: L479–85. 10.1152/ajplung.00301.2009 21148792

[19] Cultrone A, de Wouters T, Lakhdari O, Kelly D, Mulder I, Logan E, et al The NF-κB binding site located in the proximal region of the TSLP promoter is critical for TSLP modulation in human intestinal epithelial cells. Eur J Immunol. 2013; 43: 1053–62. 10.1002/eji.201142340 23310954

[20] Cho YS, Bae JM, Chun YS, Chung JH, Jeon YK, Kim IS, et al HIF-1alpha controls keratinocyte proliferation by up-regulating p21(WAF1/Cip1). Biochim Biophys Acta. 2008; 1783: 323–33. 10.1016/j.bbamcr.2007.11.017 18166158

[21] Zhao J, Du F, Shen G, Zheng F, Xu B. The role of hypoxia-inducible factor-2 in digestive system cancers. Cell Death Dis. 2015; 6: e1600 10.1038/cddis.2014.565 25590810 PMC4669763

[22] Hindryckx P, De Vos M, Jacques P, Ferdinande L, Peeters H, Olievier K, et al Hydroxylase inhibition abrogates TNF-alpha-induced intestinal epithelial damage by hypoxia-inducible factor-1-dependent repression of FADD. J Immunol. 2010; 185: 6306–16. 10.4049/jimmunol.1002541 20943999

[23] Zheng W, Kuhlicke J, Jäckel K, Eltzschig HK, Singh A, Sjöblom M, et al Hypoxia inducible factor-1 (HIF-1)-mediated repression of cystic fibrosis transmembrane conductance regulator (CFTR) in the intestinal epithelium. FASEB J. 2009; 23: 204–13. 10.1096/fj.08-110221 18779379 PMC2626614

[24] Morote-Garcia JC, Rosenberger P, Nivillac NM, Coe IR, Eltzschig HK. Hypoxia-inducible factor-dependent repression of equilibrative nucleoside transporter 2 attenuates mucosal inflammation during intestinal hypoxia. Gastroenterology. 2009; 136: 607–18. 10.1053/j.gastro.2008.10.037 19105964

[25] Stavik B, Espada S, Cui XY, Iversen N, Holm S, Mowinkel MC, et al EPAS1/HIF-2 alpha-mediated downregulation of tissue factor pathway inhibitor leads to a pro-thrombotic potential in endothelial cells. 2016; 1862: 670–678. 10.1016/j.bbadis.2016.01.017 26826018

[26] Kim BE, Howell MD, Guttman-Yassky E, Gilleaudeau PM, Cardinale IR, Boguniewicz M. TNF-α downregulates filaggrin and loricrin through c-Jun N-terminal kinase: role for TNF-α antagonists to improve skin barrier. J Invest Dermatol. 2011; 131: 1272–9. 10.1038/jid.2011.24 21346775 PMC8609659

