# Supplementary figures and images for: Hypoxia inhibits TNF-α-induced TSLP expression in keratinocytes
Source: PLoS One. 2019 Nov 4;14(11):e0224705. doi: 10.1371/journal.pone.0224705 (PMC6827910; doi:10.1371/journal.pone.0224705)

Supplement 1

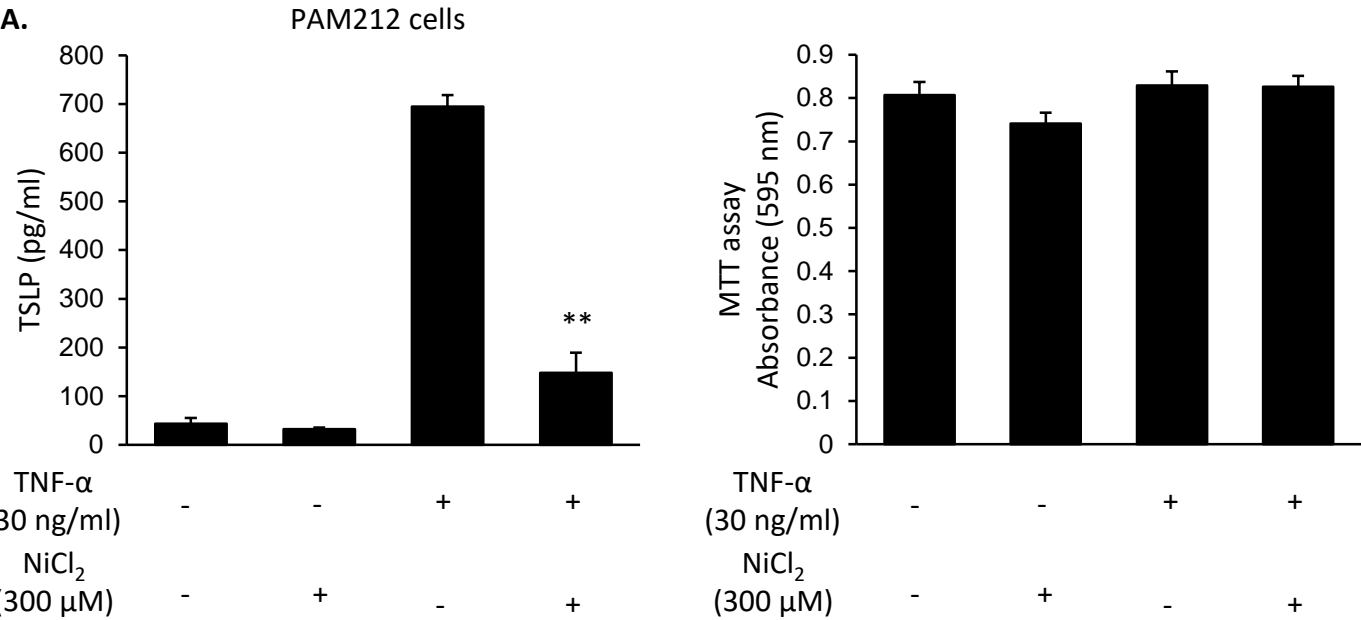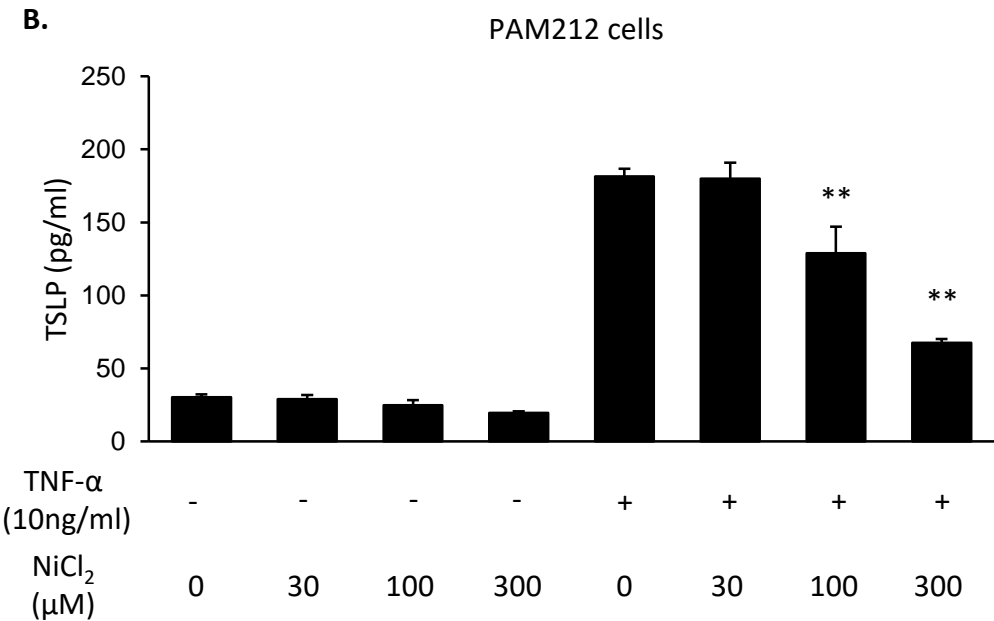

Supplement: S1 Fig — PAM212 cells were incubated for 24 h in medium containing TNF-α in the presence or absence of NiCl2 at the indicated concentrations. The concentrations of TSLP in the medium were determined by Enzyme-linked immunoassay (ELISA) and cell viability was determined by the MTT assay. Data are indicated as means ± SEM from 4 samples. Statistical significance: ** p < 0.01 vs. corresponding TNF-α control. (PDF) [file pone.0224705.s001.pdf]

Supplement 2

A.

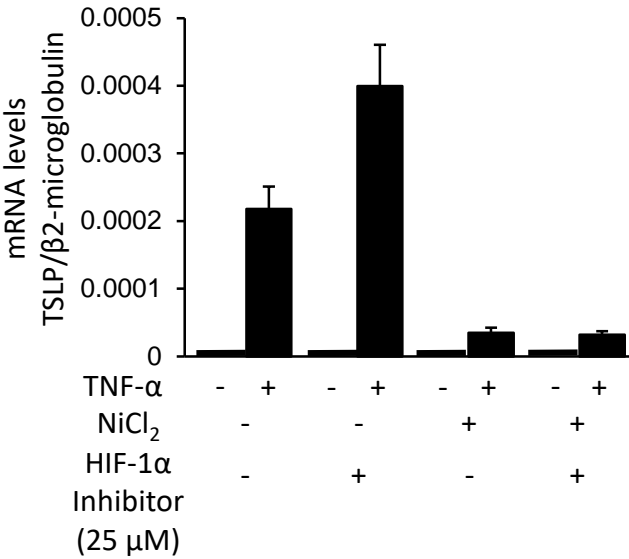

B.

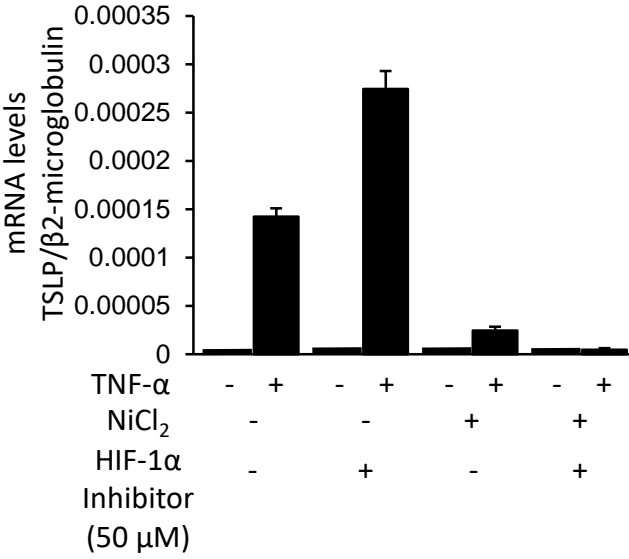

Supplement: S2 Fig — HaCaT cells were pretreated with HIF-1α inhibitor (25 μM, 50 μM) and NiCl2 (1 mM) for 8 h and stimulated with TNF-α (100 ng/ml) for 2 h. Data are indicated as means ± SEM from 3 samples. (PDF) [file pone.0224705.s002.pdf]
