# Supplementary material for: Hypoxia inhibits TNF-α-induced TSLP expression in keratinocytes
Source: PLoS One. 2019 Nov 4;14(11):e0224705. doi: 10.1371/journal.pone.0224705 (PMC6827910; doi:10.1371/journal.pone.0224705)
Supplement: S3 Fig — Putative HRE site, 5′-GACATG-3′, is indicated. HRE was searched by the TRANSFAC program (Match -1.0 Public). (PDF) [file pone.0224705.s003.pdf]

Supplement 3

HRE

-71 -62 -57

GAAGAGAAT**GACATG**GTAGAAAATCATTGGCCTAGGAGAAAAGAGCCCGTAGGCGTTTAGGT

GTTATATAGTGCAGCCAGAAAGCTCTGGAGCATCAGGGAGACTCCAACTTAAGGCAACAGCAT

GGGTGAATAAGGGCTTCCTGTGGACTGGCAATGAGAGGCAAAACCTGGTGCTTGAGCACTGG

CCCCTAAGGCAGGCCTTACAGATCTCTTACACTCGTGGTGGGAAGAGTTTAGTGTGAAACTGG

GGTGGAAT

+185
